# Physiological and Psychometric Assessment of a Multimodal e-Motion Training in Women

**DOI:** 10.3390/healthcare14101270

**Published:** 2026-05-07

**Authors:** Andrea Chellini, Nicola Gerbi, Simone Schinco, Marco Iosa, Giovanni Morone, Claudia Salera

**Affiliations:** 1 Behaviour & Movement, 50142 Firenze, Italy; kellini72@yahoo.it; 2 Embodimetria Center, Behaviour & Movement, 56083 Ponsacco, Italy; gerbi.nicola@gmail.com; 3 Embodimetria Center, Behaviour & Movement, 71211 Foggia, Italy; schincosimone@gmail.com; 4 Department of Psychology, Sapienza University of Rome, 00185 Rome, Italy; marco.iosa@uniroma1.it; 5 IRCCS Santa Lucia Foundation, 00179 Rome, Italy; 6 Department of Life, Health and Environmental Sciences, University of L’Aquila, 67100 L’Aquila, Italy

**Keywords:** wellbeing, wellness, stress

## Abstract

**Background/Objectives**: Physiological and psychological health could be altered in modern societies due to stressful environments and activities. A multimodal training based on nutrition, physical activity, breathing and hugging was proposed for improving physiological parameters in a group of women compared to a control group. **Methods**: 33 women (41 ± 15 years old) were enrolled, divided in two groups, and assessed before and after 6 weeks. Heart rate parameters, superficial adipose tissue (SAT), and trunk rotational range of motion (ROM) were measured. Stroop test and Forward Digit Span Task (FDST) were administered, in order to assess cognitive functions. **Results**: The trained group showed a significant improvement in the very low-frequency domain of heart rate variability (*p* = 0.002), a reduction in the high-frequency domain (*p* = 0.003), an improvement in the number of women with a physiological SAT (*p* = 0.014), and an improvement in memory (*p* = 0.005). In the control group, only improvements in the performances of memory (*p* = 0.029) and attention (*p* = 0.004) at Stroop test were observed. **Conclusions**: Changes in cardiac parameters and physiological level of adipose tissue showed significant variations following the multimodal training. For attention and memory, the improvements were observed also in the control group and could be related to a learning effect of the tests.

## 1. Introduction

In modern societies the stressful role overload may represent a threat to psychological health and to cognitive and motor wellbeing. This could be particularly challenging in women combining a working career and maternity [1,2]. Neuroergonomic studies have shown that cognitive load and coordination demands in fast-paced tasks are associated with measurable changes in brain activity and efficiency, highlighting the impact of real-world stressors on neural functioning [3]. The following are among the suggested non-medical interventions for favoring wellbeing enhancement: nutritional interventions [4], physical exercises [5], and nasal breathing training [6].

From a nutritional point of view, gluten-free diets were suggested also in healthy subjects because they are effective in significantly improving red blood count, hemoglobin, total cholesterol, high-density lipoprotein parameters, and psychological body satisfaction (despite being associated with increased social insecurity and reduction of magnesium levels) [7]. Moreover, the assumption of magnesium is fundamental as it is a micronutrient involved in many physiological pathways and is essential for the maintenance of normal cell and organ function [4]. Furthermore, due to its involvement in energy metabolism, it helps with muscular contraction and relaxation [8]. For instance, a supplement of oral magnesium bisglycinate chelate significantly reduced both frequency and intensity of cramps in pregnant women [9].

Concerning physical activity and programmed exercises, these activities have been widely recommended to reduce perceived stress in women, although some controversial results were reported and a meta-analysis did not confirm the efficacy of this kind of intervention [10].

Finally, nasal breathing enhances the activity and connectivity of brain regions associated with the default mode network in healthy subjects, improving attention and self-cognitive skills [6]. Furthermore, nasal breathing can affect different olfactory cortical and subcortical regions, which may be essential in transitioning from unconsciousness to wakefulness [6]. Conversely, oral breathing can lead to hypertrophy of the accessory inspiratory muscles, which impede diaphragmatic movement because of their reduced mobility and lack of coordination with the abdominal muscles [11]. Nasal breathing is also effective in reducing hyperventilation [12] and blood pressure, and improving heart rate variability (HRV) metrics, especially for reducing the low-frequency component of HRV [13].

According to the above literature, the concept of embodied mind strictly connects brain and body functions [14], and, under the assumption that it is possible to assess embodimetrics [15], recent biomechanical approaches also highlight how quantitative brain metrics are crucial for understanding functional outcomes under stress conditions [16]. In the present work we planned an observational study to analyze the quantitative effects of a multimodal non-medical intervention focused on an emotional state called e-Motion training, which is based on: physical exercises, gluten free nutrition, magnesium bisglycinate assumption, and a hugging task (hugging a familiar person at least one time per day), because hugs are associated with the attenuation of negative mood [17] and with the increment of resilience [18]. The effects of embracing could be seen in different studies. Increasing warm touch among couples showed to have a beneficial influence on multiple stress-sensitive systems, with a release of oxytocin and reduction in cortisol [19]. The effect of hugs on reducing stress was particularly evident in women, more than in men [20].

The aim of this study was to test the e-Motion training in a group of Italian women and assess the changes on their wellbeing in terms of physio- and psychological parameters and superficial adipose tissue (SAT), which is a well-organized adipose layer of hypodermis that is often increased in stressed conditions [21]. The study will provide new insights concerning quantitative assessment of integrated multimodal lifestyle interventions combining physiological, cognitive, and emotional components in healthy women. This study was conducted according to the TREND statement for non-randomized evaluations of behavioral interventions [22].

## 2. Materials and Methods

### 2.1. Participants

Thirty-three women have been enrolled in this study. Inclusion criteria were: female gender, employed status, being a mother. Exclusion criteria: presence of pathological conditions. Their mean age was 41 ± 15 years old. Seventeen women participated in the e-Motion training for 6 weeks and were assessed before and after the training, as detailed below, with the aim to combine multiple dimensions such as motor, cognitive and emotional components. Their data were compared with those of other sixteen women assessed before and after a similar period of 6 weeks in which this second group did not perform any training. The two groups were matched for age (*p* = 0.127), and both groups involved employed mothers, recruited in January 2025. The consent form for participation was distributed to all participants and signed.

### 2.2. Intervention

The intervention lasted 6 weeks (January–March 2025) and included the following components: nasal breathing training (aimed at promoting optimal respiratory function and reducing reliance on oral breathing), postural self-extension exercises (performed in a gravity-assisted standing position, with relaxed knees and barefoot, practiced daily at home to encourage natural alignment and proprioceptive stimulation, maintaining the posture for at least 3 min per day), lower limb strength and cardiovascular activation (a daily routine of 10 sit-to-squat movements along with walking up and down one flight of stairs with a minimum of 15 steps, once per day), cognitive stimulation (engagement in one crossword puzzle per day to support sustained attention), enhancement of emotional connection (a recommendation to embrace a family member at least once daily, as a mean of oxytocin release), a strict gluten-free diet (aimed at reducing potential inflammatory responses and improving gastrointestinal and systemic health), evening supplementation with magnesium bisglycinate (dose: 300 mg, with the aim to support relaxation, neuromuscular function, and sleep quality). Adherence to the protocol was checked with an app in which, every day, the enrolled women flagged the performed activities. We also enrolled a control group, monitored for the same period without receiving any particular intervention. Each daily session lasted about 30 min.

### 2.3. Assessment

We performed an embodimetric assessment based on the combination of measures related to cardiac, cognitive and motor variables [15]. This protocol was previously validated, and it measures heart rate variability, cognitive Stroop task performance, and trunk mobility.

Participants sat on a comfortable table wearing a sensorized trunk band (Beyond Inertial, Motustech, Rome, Italy), embedding an inertial measurement unit with a triaxial accelerometer, a triaxial gyroscope, and a magnetometer, as well as electrodes for assessing the heart signal. The assessment protocol involved measuring heart rate variability at baseline for 5 min. Subsequently, participants were asked to perform trunk rotations with their arms flexed on the trunk on the left and right side. The protocol was detailed elsewhere [15], but it allows to extract the frequency analysis of heart rate variability (HRV), root mean square of successive differences (RMSSD), angle phase, and trunk range of motion (ROM, computed by summing the rotation towards left and right).

The primary outcome of this study was HRV, all the other measures were considered as secondary outcomes.

The measurement of heart functions is often used for the assessment of physiological wellbeing [23]. These parameters represent the ability of the heart to respond to various perturbations and reflect the state of the autonomic nervous system, especially in stressful or psychologically demanding situations [24]. The high-frequency (HF) spectrum (0.15–0.4 Hz) of HRV corresponds to vagally mediated modulation associated with respiration (parasympathetic activity) [25]. The low-frequency (LF) spectrum (0.04–0.15 Hz) corresponds to the baroreflex control of heart rate and reflects mixed sympathetic and parasympathetic modulation of HRV, depending on the circumstances of the assessment, and is increased in anxious and stressful conditions. HRV analysis was performed using proprietary software embedded in the acquisition system (Beyond Inertial, Motustech), which computes frequency-domain parameters through standard spectral analysis (Fast Fourier Transform). Activity in the very low-frequency (VLF) spectrum (0.01–0.04 Hz) can provide another index of sympathetic and parasympathetic interaction indicating reduced resilience to stress [26]. It has been suggested that the VLF band of HRV represents the slow recovery component after mental stress tasks [27]. We also measured the phase angle, that is a measure of the asymmetry in HRV [28], and the RMSSD, that is one of the most widely used time-domain measures of HRV.

The SAT was assessed using echography at the abdomen level. SAT is a structured adipose layer of hypodermis that presents specific characteristics of cells and innervation; it is a continuous thin fibrous membrane that is rich in elastic fibers [21]. The frequency in women with a non-altered SAT (a layer <1.3 cm) was computed, according to previous results reporting that these values are related to a non-significant increase in subcutaneous adipose tissue [29].

Short term and working memory were assessed using the FDST. In this task the experimenter read aloud digits to the participant, and participants were instructed to listen to the sequence of the digits and then repeat them aloud in a forward manner. The task began with two digits per sequence and increased by one digit per level for a maximum of eight digits per sequence [30,31].

The five actions test (ACT-5) is part of the patented protocol (Italian patent number: 102020000017212) used to assess the psycho-physical status of the subject. It consists of an assessment of how the subject, in a supine position, is able to yield, push, reach, grab and pull a deflated ball, assigning a score from 1 to 5 according to the last action correctly performed.

Participants were also asked to perform a classical version of the Stroop task with 15 words, that was already used in subjects with daily stress [32]. The time to complete the task (Stroop Time) was recorded along with the number of errors made (Stroop Errors) [15].

### 2.4. Statistical Analysis

Data are reported in terms of mean ± standard deviation. The normality of data was assessed using Shapiro–Wilk test. If data were normally distributed, dependent (pre vs. post) and independent *t*-tests were used to compare experimental (EG) vs. control group (CG), otherwise the Wilcoxon test and Mann–Whitney u-test were used, respectively. The chi-square test was used to compare the percentage frequencies of non-altered SAT, applying the McNemar version for pre–post comparisons. Analysis of Covariance (ANCOVA) was used to compare pre–post data using covariate statistically different variables at baseline and computing the effect size (ES) using the partial eta squared. Principal Component Analysis was performed on the post–pre changes in the variables using Oblimin as the rotation method and choosing the number of eigenvalues as those on the left of the first inflection point on the scree plot. Alpha level of significance was set at 5% for all the analyses performed. All the analyses were performed using Jamovi 2.3.21 software, with the exception of the sample size calculation that was performed using GPower 3.1 on the basis of HRV (HF/LF) data, which was previously reported [15], obtaining for an effect size of 0.51, an alpha level of 5% and a test power of 80%, the need of enrolling at least 33 subjects. No missing data were observed.

## 3. Results

Thirty-three women have been enrolled in this study, and their mean age was 41.3 ± 14.6 years; mean stature: 1.63 ± 0.04 m; mean weight: 67.8 ± 12.9 kg. These three parameters were not significantly different between the two groups (*p* = 0.168, *p* = 0.447, *p* = 0.925, respectively). A 100% adherence rate to the training protocol was reported via the app by trained women, but this finding should be interpreted with caution, as self-reported data may be influenced by social desirability bias. 

Table 1 reports the data recorded pre- and post-training. At baseline, all the variables were not significantly different between the two groups, with the only exception of HRV-LF (*p* = 0.044).

In both groups we observed a significant reduction in the Stroop Time and an increment in FDST-score related to memory. Then, only the experimental group showed a significant increment in the percentage of women with a physiological SAT-level and HRV-VLF (accompanied by a significant reduction in HRV-HF). Although not significant, the increment of Thorax range and ACT5-score approached the significant threshold only in the experimental group. The wide variability among subjects limited the differences between groups at the end of the intervention to a significant difference in terms of SAT (*p* = 0.002). For taking into account the HRV-LF differences at baseline, we also performed an ANCOVA using baseline HRV-LF at baseline as covariate, but the results did not change and the interaction group × time remained statistically not significant also with this analysis (F(1,28) = 0.877, *p* = 0.357, ES = 0.030) as in the analysis reported in Table 1 (*p* = 0.152).

**Figure 1:**
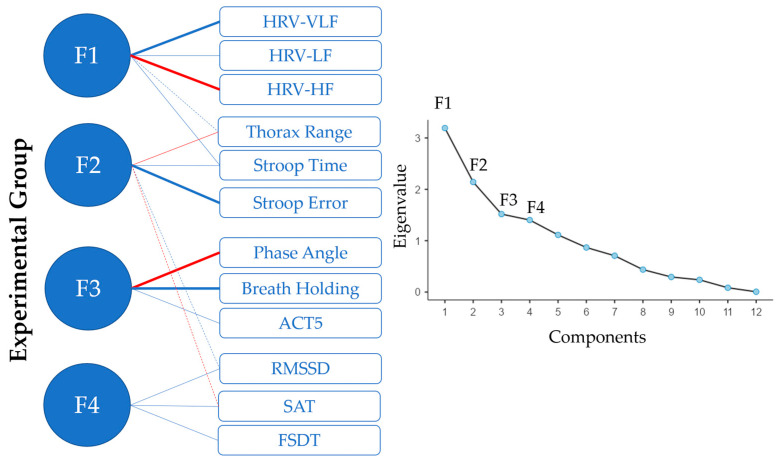
Principal Component Analysis. On the left are the factors and the load of connections (in blue if positive, in red if negative, and bolder if stronger). On the right is the scree plot identifying the eigenvalues.

Analyzing the differences in the parameters in the experimental group with Principal Component Analysis, four factors were identified (Figure 1). The first factor was related to the HRV frequencies, and also the increment of thorax range had a slight load on this factor. The second factor is related to the results of the Stroop Test. An increment of time and errors corresponded to a worsening, and it explained the negative load of thorax range and SAT changes on this factor, with a positive load of RMSSD. The third factor was related to the phase angle, breath holding and ACT5-score. Finally, the fourth factor was loaded by RMSSD, SAT and FDST-score.

The PCA was then applied to the whole sample of subjects (experimental and control groups) obtaining similar results, but with a 5-factors model: the first one related to HRV-HF (negative weight), HRV-LF and HRV-VLF; the second one to Thorax Range (negative weight), Stroop Error and Stroop Time; the third one to breath holding and ACT5, the fourth factor to RMSSD and SAT. Few differences were observed: a fifth factor formed by phase angle and FSDT (negative weight) and the Thorax Range also having an impact on factor 4.

## 4. Discussion

This study showed variations in cardiac and physiological parameters after the completion of the e-Motion multimodal six-week training, based on nutritional intervention, physical exercises, nasal breathing and familiar hugging, with increasing HRV-VLF and reducing SAT, two parameters were related with the physiological wellbeing of the participants. Quite high values of HRV-LF were noted at baseline with respect to values reported in the literature for healthy women [33], according to the presence of stress symptoms. The wellbeing alterations were also highlighted by the increased percentage of women with an altered SAT in more than half of the sample at the beginning of the study.

No changes were observed in HRV-LF, but there was a significant increment in the activity in the VLF spectrum (0.01–0.04 Hz). HRV-VLF has been suggested to reflect sympathetic influences on heart rate during apnea; however, this association should be interpreted with caution, as VLF power likely reflects multiple physiological mechanisms and cannot be considered a specific index of sympathetic activity alone. It has been suggested that the VLF band of HRV represents the slow recovery component after mental stress tasks [27]. 

Being expressed in percentage of the total frequency spectrum of HRV, the increment of VLF should be accompanied by a reduction in the other fields, and it was observed that it occurred in high frequencies (HF), but not in low frequencies (LF).

One of the most important results of this study was that the percentage of women with a physiological level of SAT significantly increased only in the experimental group, from about 47% to 82%. Similar results for SAT levels were already reported in a multimodal training, for example, treatment with exenatide combined with a change in diet and physical activity, resulted in a reduction in SAT in women with a diagnosis of lipedema, at the levels of the lower limbs, abdomen and upper limbs [34].

The improvements in Stroop test occurred in both groups and it could be related to familiarization with the task and learning effects at the second assessment. Similarly, the memory score assessed with the FDST improved in both groups, probably for the same reasons.

A trend of increment was noted also for the Thorax range and ACT5-score, approaching a statistically significant change only in the experimental group. Thorax range of motion improved on average by 8° in the experimental group, a trend that was not statistically significant but associated with the factor F1 related to cardiac activity, as found in the Principal Component Analysis. In a previous study [15], it was found that a similar intervention focusing on the perception–action link significantly improved the trunk rotational ROM by 17°.

Analyzing the differences in the parameters in the experimental group with Principal Component Analysis, four factors were identified. The first factor was related to HRV frequencies, and the increase in thorax range had a slight load on this factor. Therefore, it could be identified as a factor related to cardiological functioning. The relationship between cardiac variables and thorax movements has already been highlighted in scientific literature [15]. The second factor was related to the results of the Stroop Test. This factor could be related to cognitive effort, which is also affected by other parameters. Increased completion time and error rates on the Stroop Test corresponded to a worsening of performance, explaining the negative load of thorax range and SAT variations on this factor, along with a positive load of RMSSD. The third factor was related to phase angle, breath holding and ACT5-score. Finally, the fourth factor was loaded by RMSS D, SAT and FDST. Higher values of RMSSD correspond to greater vagal tone and typically better cardiovascular and autonomic health, whereas lower values relate to reduced parasympathetic activity, often associated with stress, fatigue and overtraining. The positive values of RMSSD were associated with physiological SAT levels and positive values for FDST memory scores.

It is important to highlight that the use of an app for controlling the adherence assured that all women in the experimental group followed the protocol; on the one hand, but on the other hand it could have increased their motivation. Apps started to be useful tools for simple digital health systems in many different protocols [35,36].

This study has some limitations. The first one is the reduced sample size, which was calculated according to the primary outcome (HRV), but may be limited considering the secondary outcomes, despite some results being statistically significant. The reduced sample size suggests caution, especially in the interpretation of the results of the Principal Component Analysis. Another limitation is the absence of psychological measures of parameters such as stress, anxiety and depressive symptoms. Moreover, this is an observational study in which the parameters of subjects participating in the training were compared with the parameters recorded in a control group; however, it was not a randomized controlled trial because the allocation occurred by choice and not randomly.

Another limitation of our study is the use of the five actions test that is a patented test, but its psychometric characteristics, such as convergent validity and test–retest reliability, should be investigated. Therefore, results should be interpreted with caution, as the reliance on the presented test should be further evaluated in future studies. Finally, multimodal training prevents us from knowing the specific effect of each of the individual components; however, previous studies already reported some suggestions about the efficacy of the single interventions merged in the multimodal approach tested in this study.

## 5. Conclusions

Despite these limits, after the completion of the e-Motion multimodal training, the experimental group showed variations in selected physiological parameters and superficial adipose tissue in a sample of healthy women, which are parameters related to wellbeing.

## Figures and Tables

**Table 1:** Means ± standard deviations of the assessed parameters pre- and post-intervention are reported. Within-group *p*-values are obtained by paired *t*-tests if data were normally distributed or by Wilcoxon tests (indicated with * on *p*-values) for non-normally distributed data. Between-group values (EG vs. CG columns) were obtained by unpaired *t*-tests if data were normally distributed or by Mann–Whitney u-tests (indicated with * on *p*-values) for non-normally distributed data. SAT was reported as the percentage relative frequency of women with a non-altered values and compared using chi-square tests (indicated with ** on *p*-values), with McNemar correction for pre–post comparisons.

Variables	Experimental Group			Control Group			EG vs. CG	
	Pre	Post	EG *p*-Value	Pre	Post	CG *p*-Value	*p*-Value Pre	*p*-Value Post
HRV-VLF	21 ± 6	25 ± 6	0.002	23 ± 6	26 ± 6	0.177	0.287	0.573
HRV-LF	34 ± 6	34 ± 7	0.920	30 ± 6	31 ± 5	0.248	0.044	0.152
HRV-HF	46 ± 9	40 ± 9	0.003	48 ± 10	43 ± 9	0.133	0.850 *	0.368
RMSSD	52 ± 7	52 ± 7	0.792	53 ± 9	52 ± 6	0.752 *	0.925 *	0.609
Phase Angle	6.7 ± 0.8	6.6 ± 0.8	0.388	6.6 ± 0.5	6.6 ± 0.5	0.628	0.624	0.850 *
SAT (%)	47.1%	82.4%	0.014 **	31.3%	25.0%	0.317 **	0.353 **	<0.001 **
Stroop Time	17.0 ± 5.4	14.6 ± 4.6	0.003	20.9 ± 9.0	15.8 ± 3.5	0.004 *	0.112 *	0.184 *
Stroop Errors	0.7 ± 1.1	0.2 ± 0.4	0.106 *	0.4 ± 0.6	0.2 ± 0.5	0.203 *	0.793 *	0.453 *
FDST score	6.7 ± 0.8	7.6 ± 0.7	0.005 *	6.7 ± 1.1	7.4 ± 0.7	0.029	0.736 *	0.554
Pause	30 ± 12	32 ± 11	0.488	31 ± 12	31 ± 11	0.919	0.610 *	0.970
Thorax range	120 ± 27	128 ± 32	0.082	114 ± 28	114 ± 24	0.824 *	0.535	0.190
ACT5	2.5 ± 1.2	3.0 ± 1.2	0.066 *	2.3 ± 1.2	2.4 ± 1.5	0.999	0.457 *	0.104

## Data Availability

The data presented in this study are available on request from the corresponding author due to privacy.

## Abbreviations

The following abbreviations are used in this manuscript:

SAT	Superficial adipose tissue
ROM	Range of motion
FDST	Forward digit span task
HRV	Heart rate variability
RMSSD	Root mean square of successive differences
HF	High frequency
LF	Low frequency
VLF	Very low frequency
TCT	Time to complete the task
NE	Number of errors
ACT-5	Five action test
EG	Experimental group
CG	Control group
